# Use of 4-Fr versus 6-Fr Nasobiliary Catheter for Biliary Drainage: A Prospective, Multicenter, Randomized, Controlled Study

**DOI:** 10.1155/2017/7156719

**Published:** 2017-04-19

**Authors:** Tomofumi Tsuboi, Masahiro Serikawa, Tamito Sasaki, Yasutaka Ishii, Yoshifumi Fujimoto, Atsushi Yamaguchi, Takashi Ishigaki, Akinori Shimizu, Keisuke Kurihara, Yumiko Tatsukawa, Eisuke Miyaki, Kazuaki Chayama

**Affiliations:** ^1^Department of Gastroenterology and Metabolism, Applied Life Science, Institute of Biomedical & Health Science, Hiroshima University, Hiroshima, Japan; ^2^Department of Gastroenterology, Hiroshima Prefectural Hospital, Hiroshima, Japan; ^3^Department of Gastroenterology & Hepatology, JA Hiroshima General Hospital, Hiroshima, Japan; ^4^Department of Gastroenterology, National Hospital Organization, Kure Medical Center and Chugoku Cancer Center, Kure, Hiroshima, Japan; ^5^Department of Gastroenterology, National Hospital Organization Higashihiroshima Medical Center, Higashihiroshima, Japan

## Abstract

*Background and Aim.* Endoscopic nasobiliary drainage (NBD) effects according to diameter remain unclear. We aimed to assess the drainage effects of the 4-Fr and 6-Fr NBD catheters.* Methods.* This prospective, multicenter, randomized, controlled study was conducted at Hiroshima University Hospital and related facilities within Hiroshima Prefecture. Endoscopic retrograde cholangiopancreatography (ERCP) in 246 patients revealed acute cholangitis, obstructive jaundice, and/or extrahepatic cholestasis; 4-Fr or 6-Fr NBD catheters were randomly allocated and placed in these patients. The primary endpoint was the efficacy of NBD based on the technical success rate and clinical success (rates of change in blood test and amount of bile output). Secondary endpoints included the spontaneous catheter displacement rate and nasal discomfort.* Results.* The technical success rate and clinical success did not differ significantly between groups. No spontaneous catheter displacement was noted in either group. Nasal discomfort due to catheter placement was significantly lower in the 4-Fr group versus the 6-Fr group (24 h after ERCP: 2.4 versus 3.5 cm, *P* = 0.005; 48 h after ERCP: 2.2 versus 3.1 cm, *P* = 0.01).* Conclusion.* The 4-Fr NBD catheter was not inferior to 6-Fr NBD catheter in terms of clinical success; the 4-Fr NBD catheter was useful to reduce nasal discomfort.

## 1. Introduction

The 3 methods of drainage treatment for acute cholangitis and obstructive jaundice include endoscopic drainage, percutaneous drainage, and surgical drainage [1]. A randomized controlled trial (RCT) that compared endoscopic drainage and surgical drainage for cholangitis showed that endoscopic drainage was safer and more effective in terms of the mortality rate and the complication rate [2]. Although no RCT has compared endoscopic drainage and percutaneous drainage in terms of invasiveness, frequency of complications, and length of hospital stay, endoscopic drainage is often considered as the preferred choice [3, 4]. In particular, endoscopic drainage can be performed via endoscopic nasobiliary drainage (NBD) and endoscopic biliary stenting (EBS). Although there is not any study to refer to with a head to head comparison of NBD to EBS with regard to clinical effectiveness, in East Asian countries such as Japan, NBD is preferred to EBS. The reasons for such a preference include monitoring the amount and quality of drained bile, identifying causative bacteria in cholangitis, and facilitating pathological examination. In contrast, NBD-induced obstructive pancreatitis, nose/throat discomfort, unacceptable esthetics, and self-removal by the patient are problems associated with NBD. We have previously reported that catheter diameter may be related to the occurrence of obstructive pancreatitis and throat discomfort [5]. At present, catheters of various sizes are available for NBD, ranging from 5-Fr to 7.5-Fr; however, the drainage results according to catheter size remain unclear. Here, we performed a multicenter RCT to verify whether the drainage results associated with the use of the 4-Fr NBD catheter are equivalent to those associated with the use of the 6-Fr NBD catheter (a widely applied catheter).

## 2. Materials and Methods

### 2.1. Study Design

In this multicenter, prospective, randomized controlled study, we compared and evaluated the clinical effectiveness of the 4-Fr catheter and the 6-Fr catheter in cases undergoing NBD.

The study was conducted at Hiroshima University and 11 affiliated hospitals.

The study protocol is available at the University Hospital Medical Information Network (UMIN) Clinical Trial Registry (UMIN000012677).

The protocol conformed to the Declaration of Helsinki and was approved by the institutional review board of Hiroshima University Hospital. Enrollment began in July 2013 and was completed in July 2015.

### 2.2. Patient Selection

We enrolled patients with obstructive jaundice, with acute cholangitis requiring biliary tract drainage, without jaundice, and with suspected cholestasis based on blood and imaging tests (bile duct diameter ≥ 10 mm on ultrasonography, computed tomography, and/or magnetic resonance imaging); all enrolled patients provided written informed consent for participation in the study.

Patients were excluded from the study if they were aged < 20 years; pregnant, breastfeeding, or possibly pregnant; had a history of endoscopic biliary sphincterotomy (EST) or endoscopic papillary balloon dilatation (EPBD); had acute pancreatitis or a serum pancreatic enzyme level ≥ 3 times the upper limit of normal prior to ERCP (pancreatic amylase or lipase values were preferred); had a history of sphincter of Oddi dysfunction; had pancreas divisum and malfusion of the pancreaticobiliary ducts; had a history of upper gastrointestinal tract reconstruction involving techniques other than Billroth 1 reconstruction, postoperative anatomy hindering endoscope from reaching the papilla; had undergone duodenal papilla procedures such as EST, EPBD, and papilla precutting; had prior or concurrent endoprosthesis other than the NBD used in the study (patients with concurrent biliary stents or multiple indwelling NBD catheters); were undergoing drainage of the right bile duct branch; had liver failure; or were allergic to polyurethane or ERCP contrast medium.

### 2.3. Randomization

A system was constructed on a server managed by a network management company, and each facility—which had been randomly allocated a specific catheter size to be used—entered data via the Internet. The facilities set obstructive jaundice as a stratification factor. The allocated catheter size was not communicated to the patient.

### 2.4. Sample Calculation and Statistical Analysis

We determined that the 4-Fr NBD catheter is as effective as the conventionally used 6-Fr NBD catheter. The efficacy of 4-Fr and 6-Fr NBD catheters is reportedly 92%. When the noninferiority limit was set to 10% (i.e., to verify whether the efficacy of the 4-Fr catheter is not <10% lower than that of the 6-Fr catheter), the *α* error was set to 0.05, and the power was set to 0.8, we found that each group would require 92 patients. Considering an expected 20% dropout rate, we concluded that 220 patients would be required overall (110 patients in each group); the 20% dropout rate accounted for the exclusion of certain patients after registration according to the exclusion criteria.

The Mann–Whitney *U* test was used to analyze the continuous variables, whereas the chi-squared test or Fisher's exact test was used to analyze categorical data (depending on the suitability). A *P* value of <0.05 was considered statistically significant.

### 2.5. NBD Catheter (Figure 1)

A 4-Fr or 6-Fr polyurethane NBD catheter (260 cm; Gadelius Medical KK, Tokyo, Japan) was used for ERCP. The 4-Fr catheter had an internal diameter of 1.05 mm and an external diameter of 1.30 mm, whereas the 6-Fr catheter had an internal diameter of 1.30 mm and an external diameter of 2.00 mm. The ends were hooked to facilitate bile duct insertion in the lateral segment of the liver (B2 or B3). The catheters were tapered at a distance of 10 mm from the tip; 17 side-holes (0.8 mm in diameter) were created at 5 mm intervals in a spiral pattern, spanning 10–90 mm from the tip. The duodenal portion of both the 4-Fr and 6-Fr catheters was shaped into a loop.

### 2.6. Placement of the NBD Catheter

Intravenous anesthesia (midazolam or flunitrazepam or diazepam) was used for all patients during ERCP. The anesthesia was adopted by the physician. The deep bile duct cannulation method was not permitted, although bile duct cannulation was performed using the methods normally adopted at each facility. After bile duct cannulation, a guidewire was introduced into B2 or B3. Intraductal ultrasonography (IDUS), brushing cytology, and bile duct biopsy were then performed as needed, and the NBD catheter was placed carefully. A nasopancreatic drainage (NPD) tube or pancreatic stent was placed in patients considered to be at high risk of pancreatitis, at the discretion of the physicians at each facility.

### 2.7. Protocol

Informed consent was obtained for ERCP and for study participation. After consent was provided for study participation, the patient's information was entered into the system via the Internet, and the patient was randomly allocated a specific catheter size (4-Fr or 6-Fr). Except for catheter selection, the procedure was performed as usual. At least the NBD catheter was placed for 48 hours since the NBD catheter was inserted. After 48 hours of blood test and clinical symptoms improvement, the physician selected whether the patient continues to place the catheter or remove it. Blood was drawn within 24 hours prior to the test; at 24 and 48 hours after the test, blood was drawn again, abdominal radiography was performed, and nose/throat discomfort was evaluated on a visual analogue scale (VAS). The amount of bile output was measured by recording the time and the output volume from the start of the drainage until the removal of the catheter; the amount of bile output per hour was evaluated.

### 2.8. Study Endpoint

With regard to the primary endpoint, we compared the clinical success rate of the 4-Fr NBD catheter with that of the 6-Fr NBD catheter in patients with acute cholangitis and/or obstructive jaundice, without jaundice, and with findings of cholestasis. For the secondary endpoint, we compared nose/throat discomfort (based on the VAS score) associated with catheter placement and compared the frequency of spontaneous displacement of the catheter.

### 2.9. Subgroup Analysis

We compared the frequency of postoperative pancreatitis in patients with an indwelling NBD catheter (patients without concurrent pancreatic duct drainage with pancreatic duct stents or nasal pancreatic duct drainage) in the 4-Fr and 6-Fr groups.

### 2.10. Assessment of Blood Test Results

The rate of change in the blood test values was estimated as day 2 − day 0/day 0 × 100 (%) and was calculated for each patient. For instance, the rate of change in ALP levels was calculated as (ALP level on day 2 − ALP level on day 0)/ALP level on day 0 × 100 (%).

### 2.11. Definition of the Clinical Success Rate

#### 2.11.1. Acute Cholangitis

An effective outcome was defined as a reduction in acute cholangitis-related fever and right hypochondriac pain, as well as an improvement in white blood cell count and hepatobiliary enzyme levels within 48 hours after NBD placement. Treatment was considered to be ineffective in patients who did not show any such improvement.

#### 2.11.2. Obstructive Jaundice (T-Bil Level ≥ 3.0 mg/dL)

An effective outcome was defined as a decrease in the T-bil level to below the baseline value (pretreatment value), as well as an improvement in the imaging findings that were suggestive of cholestasis or an improvement in hepatobiliary enzyme levels within 48 hours after NBD catheter placement. Treatment was considered to be ineffective in patients who did not show any such improvement.

#### 2.11.3. Extrahepatic Cholestasis (T-Bil Level ≤ 3.0 mg/dL, Elevated Hepatobiliary Enzyme Levels, and Presence of Cholangiectasis, Clinically Indicative of Suspected Cholestasis)

An effective outcome was defined as an improvement in the hepatobiliary enzyme findings and an improvement in the cholangiectasis findings on imaging tests within 48 hours after NBD catheter placement. Treatment was considered to be ineffective in patients who did not show any such improvement.

### 2.12. Definition of PEP

As reported by Cotton et al. [6], PEP was considered in cases that developed symptoms of pancreatitis within 24 hours after ERCP or that had serum pancreatic type amylase levels more than three times the normal value at 24 hours or 48 hours after ERCP.

## 3. Results

### 3.1. Patient Characteristics

**Figure 2:**
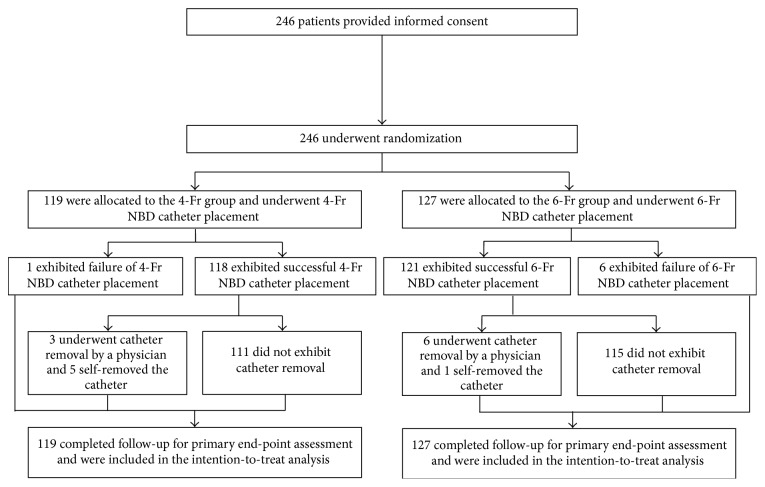
Study flowchart. Intention-to-treat analysis was performed. ERCP: endoscopic retrograde cholangiopancreatography; NBD: endoscopic nasobiliary drainage.

Informed consent was obtained from 246 patients, and NBD catheter placement was performed in all of these cases (4-Fr group, 119; 6-Fr group, 127; Figure 2). Patient background, the procedure implemented, and the time required for the procedure did not differ significantly between groups (Table 1). NBD catheter placement was successful in 118 (99.1%) of the 119 patients in the 4-Fr group and in 121 (95.3%) of the 127 patients in the 6-Fr group. The reasons for unsuccessful catheter placement in all patients included difficulty of placement due to selective bile duct cannulation. Self-removal by the patient was noted in 6 patients (4-Fr group, *n* = 5; 6-Fr group: *n* = 1), whereas the NBD catheter was intentionally removed by the physician in 9 patients [4-Fr group: *n* = 3 (2 cases were pancreatitis and 1 case was poor drainage); 6-Fr group: *n* = 6 (5 cases were pancreatitis and 1 case was poor drainage)] due to procedural accidents associated with NBD. Cases for which placement was unsuccessful, cases of self-removal by the patient, and cases for which the catheter was intentionally removed by the physician were included in the intention-to-treat (ITT) analysis.

### 3.2. Primary Outcome Measurements

The NBD clinical success rates did not differ significantly between the groups (4-Fr group: 91.6%; 6-Fr group: 89.8%; *P* = 0.78). The rates of change in the ALP levels were −18.6 ± 13.6% in the 4-Fr group and −18.3 ± 14.5% in the 6-Fr group (*P* = 0.51), whereas the rates of change in the *γ*-GTP levels were −23.9 ± 13.6% in the 4-Fr group and −25.5 ± 22.9% in the 6-Fr group (*P* = 0.10). The amount of bile output was 22.4 ± 4.4 mL/h in the 4-Fr group and 22.6 ± 3.5 mL/h in the 6-Fr group; this value did not differ significantly between groups (*P* = 0.32; Table 2). With regard to the T-bil level, 74 patients in the 4-Fr group and 84 patients in the 6-Fr group had a T-bil level ≥ 3 mg/dL. Moreover, the rate of change in the T-bil level was −37.2 ± 25.6% in the 4-Fr group and −36.3 ± 31.3% in the 6-Fr group; this value did not differ significantly between groups (*P* = 0.52; Table 3).

### 3.3. Secondary Outcome Measurements

Spontaneous displacement and kinking of the catheter were not observed in either group. Nose/throat discomfort was assessed in 216 patients, excluding cases for which NBD catheter placement was unsuccessful, cases with NPD catheter placement, cases of self-removal by the patient, and cases for which the catheter was intentionally removed by the physician. Nose/throat discomfort was 2.4 ± 2.2 cm in the 4-Fr group and 3.5 ± 2.6 cm in the 6-Fr group on day 1 (*P* = 0.005) and 2.2 ± 2.2 cm in the 4-Fr group and 3.1 ± 2.4 cm in the 6-Fr group on day 2 (*P* = 0.01); the VAS scores showed a significant reduction in the 4-Fr group (Table 2).

### 3.4. Subgroup Analysis

Four patients in the 4-Fr group and 9 patients in the 6-Fr group had concurrent NPD placement and pancreatic stent placement, whereas 115 patients in the 4-Fr group and 118 patients in the 6-Fr group underwent only NBD catheter placement.

The incidence of pancreatitis was 3.5% (4/115) in the 4-Fr group and 7.6% (9/118) in the 6-Fr group (*P* = 0.31), which was not significantly different. With regard to PEP severity, 4 mild cases were noted in the 4-Fr group, and 6 mild, 2 moderate, and 1 severe cases were noted in the 6-Fr group (Table 4).

## 4. Discussion

In 1979, Cotton et al. [6] developed NBD as a technique for transnasal bile duct catheterization during ERCP. NBD is performed for biliary drainage in obstructive jaundice, the treatment of acute cholangitis, cytodiagnosis of bile duct neoplastic lesions, and the treatment of postoperative bile leak after bile duct surgery [2, 7–10]. Catheters of various types and sizes can be used for drainage, and the selection of a specific catheter depends on the particular facility. In a previous study, we found that the use of the 4-Fr NBD catheter significantly reduced the onset of PEP and nose/throat discomfort [5]. Thus, the use of a catheter with a small diameter reduces the burden on the patient, and if such catheters have good drainage efficacy, then these smaller catheters should be the first choice for treatment. Several reports have described the effectiveness of ENBD for acute cholangitis and obstructive jaundice [11–14]; however, only a few studies have investigated the drainage effects according to NBD catheter diameter. Many studies have assessed the drainage effects of biliary stents based on the stent diameter. Sharma et al. investigated the placement and drainage results of 7-Fr and 10-Fr biliary stents in patients with cholangitis and/or obstructive jaundice [14–16] and reported that there was no significant difference between the 2 groups. Kadakia and Starnes [17] also reported that there was no significant difference in the drainage effects between 10-Fr and 11.5-Fr biliary stents [15]. However, the lengths of biliary stents and NBD catheters differ, and hence, it is difficult to conclude whether the effects of biliary stent drainage would be applicable to NBD catheter placement. Fujisawa et al. described the biliary drainage effects of NBD catheters of 5-Fr and 7-Fr diameters in cases with obstructive jaundice [18]; the authors found that the rate of reduction in bilirubin levels (day 0 − day 4/day 0 × 100) was significantly better with the 7-Fr NBD catheter than with the 5-Fr NBD catheter. However, the success rate of jaundice improvement was 98% with the 7-Fr NBD catheter and 88% with the 5-Fr NBD catheter, which was not significantly different. Furthermore, the drainage output (mL/day) did not differ significantly between the 7-Fr NBD catheter and the 5-Fr NBD catheter. Approximately 80% of the patients in that study had undergone EPBD and/or EST, and hence, these findings cannot be completely reflective of the drainage effects of an NBD catheter. The findings of the present study indicated that the rate of change in the ALP and *γ*-GTP levels and the amount of bile output (mL/h) did not differ significantly between the 4-Fr NBD catheter and the 6-Fr NBD catheter groups and that the clinical success rate did not differ significantly, either. Thus, we noted that the drainage effects of the 4-Fr NBD catheter and the 6-Fr NBD catheter are equivalent.

In the present study, we found that nasal discomfort—a major problem in NBD—was significantly reduced with the 4-Fr catheter as compared to that with the 6-Fr catheter. The VAS score for nasal discomfort with NBD catheter placement decreases as the placement time increases, suggesting that the patient becomes accustomed to the catheter. However, long-term catheter placement also increases the risk of self-removal by the patient; hence, unnecessary long-term placement should be avoided. The incidence of PEP associated with NBD catheter placement did not differ significantly between groups, although it was numerically lower in the 4-Fr NBD catheter group. In addition, the incidence of moderate or severe pancreatitis was 2.5% in the 6-Fr group, compared to 0% in the 4-Fr group. One reason for the lack of difference between groups in the incidence of PEP could be that concurrent placement of NPD and pancreatic stents was permitted in cases at high risk of developing PEP.

## 5. Conclusion

In conclusion, we found that the drainage effects did not differ between the use of the 4-Fr NBD catheter and the use of the 6-Fr NBD catheter. Moreover, we found that the 4-Fr NBD catheter was associated with reduced nasal discomfort. We believe that the 4-Fr NBD catheter should represent the first choice amongst NBD for treatment of patients with obstructive jaundice and acute cholangitis.

## Figures and Tables

**Figure 1:**
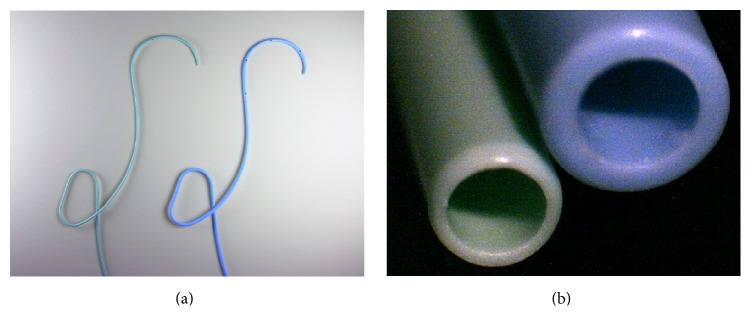
Comparison of 4-Fr and 6-Fr nasobiliary drainage catheters. (a) Left, 4-Fr; right, 6-Fr. Both have the same looped configuration. (b) Cross-sectional view. Left, 4-Fr; right, 6-Fr.

**Table 1:** Baseline characteristics and ERCP procedures.

	4Fr group	6Fr group	*P* value
	(*n* = 119)	(*n* = 127)	
Age (y), mean (SD)	70.4 (12.6)	71.5 (11.6)	0.51
Sex			
Male, *n* (%)	64 (53.4)	77 (60.6)	0.33
Female, *n* (%)	55 (46.6)	50 (39.4)	
Body mass index (kg/m^2^), mean (SD)	22.4 (4.3)	22.6 (3.5)	0.31
Indication (overlapping)			
Obstructive jaundice, *n* (%)	74 (62.2)	84 (66.1)	0.51
Acute cholangitis, *n* (%)	32 (26.9)	42 (33.1)	0.29
Extrahepatic cholestasis, *n* (%)	87 (73.1)	94 (74.0)	0.98
Diagnosis			
Choledocholithiasis, *n* (%)	39 (32.8)	38 (30.0)	0.63
Pancreatic cancer, *n* (%)	30 (25.2)	33 (26.0)	0.89
Cholangiocarcinoma, *n* (%)	21 (17.6)	22 (17.3)	0.95
Carcinoma of the papilla of Vater, *n* (%)	7 (5.9)	5 (3.9)	0.68
Gallbladder cancer, *n* (%)	5 (4.2)	5 (3.9)	0.83
Chronic pancreatitis, *n* (%)	2 (1.7)	0 (0)	
Others, *n* (%)	15 (12.6)	24 (18.9)	0.18
ERCP procedures			
Pancreatic duct injection, *n* (%)	67 (61.0)	75 (55.4)	0.66
Brushing of pancreatic duct, *n* (%)	2 (1.7)	2 (1.6)	0.66
Endoscopic pancreatic stent, *n* (%)	2 (1.7)	3 (2.3)	0.94
Endoscopic nasopancreatic drainage, *n* (%)	2 (1.7)	6 (4.7)	0.32
Biliary intraductal ultrasonography, *n* (%)	40 (33.6)	40 (31.5)	0.82
Brushing of bile duct, *n* (%)	26 (21.8)	29 (22.8)	0.85
Biopsy of bile duct, *n* (%)	3 (2.5)	2 (1.6)	0.94
Procedure time (min), mean (SD)	30.6 (14.6)	29.4 (14.1)	0.50

ERCP, endoscopic retrograde cholangiopancreatography; SD, standard deviation.

*P* value not significant for all comparisons.

**Table 2:** Outcomes.

	4Fr group	6Fr group	*P* value
	(*n* = 119)	(*n* = 127)	
Technical success rate (%)	118 (99.1)	121 (95.3)	*0.15*
Clinical success rate (%)	109 (91.6)	114 (89.8)	*0.78*
ALP (IU/L)			
Before biliary drainage	1217 (865)	1168 (908)	*0.49*
After biliary drainage	963 (673)	932 (681)	*0.51*
ALPdecreasing rate (%), mean (SD)	18.6 (13.6)	18.3 (14.5)	*0.51*
*γ*-GTP (IU/L)			
Before biliary drainage	673 (511)	679 (542)	*0.52*
After biliary drainage	504 (377)	496 (412)	*0.51*
*γ*-GTP decreasing rate (%), mean (SD)	23.9 (13.6)	25.5 (22.9)	*0.1*
Amount of bile output (mL/hour), mean (SD)	22.4 (4.4)	22.6 (3.5)	*0.32*
Displacement			
Willful removal by the doctor, *n* (%)	3 (3.7)	6 (12.0)	0.56
Self-removal by the patient, *n* (%)	5 (3.7)	1 (1.2)	0.20
Spontaneous displacement, *n* (%)	0 (0.0)	0 (0.0)	

	4Fr group	6Fr group	
	(*n* = 109)	(*n* = 107)	

VAS score of discomfort (cm)			
24 h after ERCP, mean (SD)	2.4 (2.2)	3.5 (2.6)	0.005^*∗*^
48 h after ERCP, mean (SD)	2.2 (2.2)	3.1 (2.4)	0.010^*∗*^

SD, standard deviation; VAS, visual analog scale.

^*∗*^Significant difference.

ALP: Alkaline phosphatase.

*γ*-GTP: Gamma-Glutamyl transpeptidase.

**Table 3:** In patients with obstructive jaundice.

	4Fr group	6Fr group	*P* value
	(*n* = 74)	(*n* = 84)	
Baseline serum total bilirubin (mg/dL), mean (SD)	8.3 (4.9)	9.1 (5.6)	0.41
Amount of bile output (mL/hour), mean (SD)	17.9 (9.8)	20.5 (18.1)	0.52
T-bil decreasing rate (%), mean (SD)	37.2 (25.6)	36.3 (31.3)	*0.52*

SD, standard deviation.

*P* value not significant for all comparisons.

**Table 4:** Subgroup analysis.

	4Fr group	6Fr group	*P* value
	(*n* = 115)	(*n* = 118)	
Pancreatitis, *n* (%)			
Total	4 (3.5)	9 (7.6)	0.31
Severity			
Mild	4 (3.5)	6 (5.1)	
Moderate	0 (0.0)	2 (1.7)	
Severe	0 (0.0)	1 (0.8)	
